# RADIATION DOSE OF THE EYE LENS IN CT EXAMINATIONS OF THE BRAIN IN CLINICAL PRACTICE—THE EFFECT OF RADIOGRAPHER TRAINING TO OPTIMISE GANTRY TILT AND SCAN LENGTH

**DOI:** 10.1093/rpd/ncad002

**Published:** 2023-01-23

**Authors:** Jeremias Tarkiainen, Miia Nadhum, Annele Heikkilä, Irina Rinta-Kiikka, Atte Joutsen

**Affiliations:** Faculty of Medicine and Health Technology, Tampere University, Tampere, Finland; Faculty of Medicine and Health Technology, Tampere University, Tampere, Finland; Department of Medical Physics, Medical Imaging Center, Pirkanmaa Hospital District, Tampere, Finland; Faculty of Medicine and Health Technology, Tampere University, Tampere, Finland; Department of Medical Physics, Medical Imaging Center, Pirkanmaa Hospital District, Tampere, Finland; Department of Radiology, Medical Imaging Center, Pirkanmaa Hospital District, Tampere, Finland; Department of Medical Physics, Medical Imaging Center, Pirkanmaa Hospital District, Tampere, Finland

**Keywords:** Gantry tilt, eye lens, lens irradiation, supraorbitomeatal line, scan length optimisation

## Abstract

Lenses are always exposed to radiation in brain computed tomography (CT) scans. However, the lens dose can be reduced by excluding lens from scanning area by optimising gantry tilt and scan length. The object of this study is to retrospectively analyse if the optimisation by gantry tilt and scan length have been adequate in the CT scan of the brain, and to prospectively analyse the effect of radiographer training to the quality of the CT examinations. This study was conducted in two parts. In all, 329 brain CTs performed in the Tampere University Hospital from 2017 to 2019 were revised retrospectively. The prospective part included 51 brain CT studies conducted in October 2021. Dose to the eye of the lens was modelled using CT-Expo using zero-degree beam angle and scan lengths to expose the lens either to the primary or scattered radiation. Non-zero gantry tilt had been used in a large proportion of the CT examinations in the retrospective setting, 84.8%. However, the lenses were successfully excluded from the scan area in only 1.8% of the examinations. In the prospective part, the gantry tilt was used in 98% of the studies and the proportion of successful examinations rose from 1.8 to 11.8%. The lens dose decreased significantly when the eyes were excluded from the imaging area. The modelled lens dose in the large retrospective part was 25.9 mGy (17.8–49.2 mGy) when the eyes were included and 1.5 mGy (0.4–1.9 mGy) when the eyes were excluded. The lens dose was similar in the small prospective part. Despite the gantry tilt is widely used, unnecessary lens irradiation occurs extensively because of suboptimal gantry tilt and scan length. The training of radiographers reduces the radiation exposure to the lens by more optimal gantry tilt and scan length.

## Introduction

Computed tomography (CT) is an essential diagnostic modality as demonstrated by the annually increasing number of performed examinations^(1)^. Although the radiation doses of CT examinations have decreased because of the developments in hardware and reconstruction algorithms, CT is still the main source of radiation exposure from medical imaging devices^(2)^. Brain imaging constitutes up to 40 and 65% of the performed CT examinations for adults and young people, respectively^(3, 4)^, presenting the justification for their optimisation (i.e. reducing patient dose).

**Figure 1:**
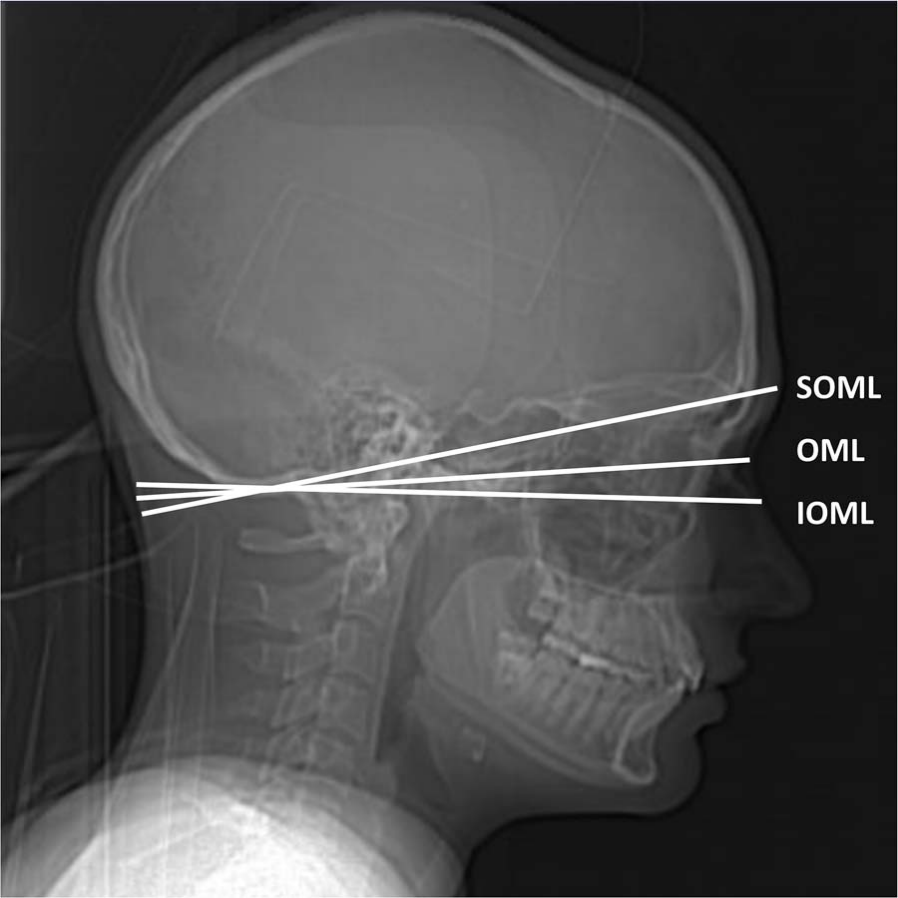
The three anatomical reference lines used to fit the gantry tilt angle to the anatomy. These reference lines were used in the present study to classify the examinations: SOML (Class A), OML (Class B) and IOML (Class C).

Furthermore, the lens of the eye is one of the most radiation-sensitive organs^(5)^, with increased exposure levels potentially causing a cataract^(6)^—a leading cause of blindness and visual disability worldwide^(7)^. Radiation-induced cataracts are currently classified as deterministic with the threshold dose of 500 mSv^(5, 8)^. However, some studies show signs that cataract formation may be associated with radiation doses lower than that^(9–12)^, whereas other studies show that the eye lenses may not be as radiosensitive as originally thought^(13, 14)^. Despite the controversies related to the amount of radiation dose required to cause cataracts, brain CT examinations should be optimised in terms of radiation exposure and the field-of-view to limit the exposure of lenses. Currently, the radiation dose of lenses can also be reduced by adapting organ-based dose modulation^(15)^, or optimising gantry tilt angle. When the gantry tilt angle is aligned with the supraorbitomeatal line (SOML, Figure 1), the lens is not exposed to the primary beam and the radiation dose is reduced by 75–93%^(16–19)^. However, Omer *et al*.^(20)^ noted that imaging protocols and lack of training can cause variations in the radiation doses of the lenses.

The aim of this study was to investigate and optimise the radiographer’s usage of gantry tilt angle and scan length selection to reduce the radiation doses of lenses in head CT examinations. First, the imaging protocols were reviewed and radiographers were trained to apply optimal gantry tilt angle and scan length. Second, the irradiated volumes and radiation doses of lenses were investigated before and after the training to assess the impact of the training.

## Materials and methods

The study was conducted in the Tampere University Hospital, where 15 000 brain CT examinations are performed yearly. This study included patients that were scanned using a Revolution GSI scanner (GE Healthcare, Milwaukee, WI, USA), which features a tilting gantry (±30° max tilt, 0.5° increments). The study had retrospective and prospective parts. The inclusion criteria for the retrospective part were the following: (1) patient scanned using the Revolution GSI scanner during 2017–19, (2) patient age between 16 and 40 years, (3) no indication for intentionally scanning the orbital area, (4) axial images available and (5) no dose increasing implants. Finally, 329 brain CT scans were eligible for the retrospective analysis. Of those, eight patients had a shunt and one had deep brain stimulation electrodes. Their volume CT dose index (CTDI_vol_) did not differ from the rest and thus were included. The collected data from patients included: age, sex, CTDI_vol_, dose length product (DLP) and gantry tilt angle. The results were assessed, and a radiographer training was tailored to address the issues found.

The effect of the training of the radiographers on imaging optimisation was examined by further analysis. The radiographers were trained by giving instructions about imaging optimisation principles in the brain CT scan. The follow-up analysis consisted of 51 patients who underwent CT examination in October 2021. The inclusion criteria for the prospective part were the same as for the retrospective part, except that there was no age criterion in the follow-up analysis, because only few patients under 40 years were scanned during the follow-up period and thus, the sample size would have been too small. The follow-up CT studies were analysed according to the same principles as in the first group of patients.

**Figure 2:**
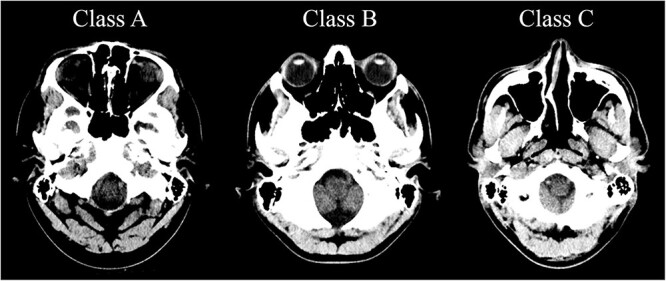
The lowest image in the stack was used to classify the examination. From left: Class A—lens exposed only to scattered radiation, Class B—lens partially exposed to primary radiation and Class C—lens fully exposed to primary radiation.

In optimal brain CT scan, the lens is not exposed to the primary radiation. Three different categories were used to classify if the gantry tilt and scan length were optimal. In Class A, the scan area is fitted to SOML. In Class C, the scan area is fitted to IOML or below. The Class B is between Classes A and C where the scan area is fitted around OML (Figure 1). The lowest image in the CT image stack was used to classify the study (Figure 2).

The eye lens doses were modelled by using CT-Expo v. 2.7^(21)^ (SASCRAD, Buchholz, Germany). The CT-Expo is implemented in Microsoft Excel using Visual Basic for Applications. The application can be used for assessing gender- and age group-related whole-body- and organ-specific radiation doses delivered to patients undergoing CT examinations. CT-Expo has been validated for a variety of CT scanners^(22)^ including the GE Revolution GSI scanner used in the present study. CT-Expo does not support dose modelling with tilted gantry and therefore the eye lens doses were modelled using 0-degree beam angle. The CT-Expo phantom exposure volume selection is made in *Z*-direction in one centimetre steps, which introduces uncertainties in Class B. After considering the limitations eye-lens doses were modelled in only Classes A and C with the *Z*-direction volume caudal limit above the eyes and below the eyes.

The modelling parameters for the dose calculation were collected from the brain scans’ DICOM headers: X-ray tube voltage, tube current in the image where the lens is visible (Class C) or the lowest image in the stack (Class A), revolution time, total collimation width, operating mode: axial mode with no section overlap, slice thickness and patient’s sex. Female patients were modelled using the EVA adult female and male patients using the ADAM adult male phantom. The modelling scan length was selected such that it covered the entire skull of the phantom to ensure that the whole lens was inside the scanning area (Class C) or outside the scanning area (Class A). It was assumed that the calculated lens exposure was not significantly altered by whether the scan length in Class C was extended to just below the eye or to the base of the mandible. The modelled lens equivalent doses were compared with the lens doses found in the literature. Median and range were calculated for continuous variables and compared with Kruskal–Wallis *H* or Mann–Whitney *U* tests. Categorical variables are represented as proportion percentages. The data of this study are openly available in Mendeley Data at doi: https://doi.org/10.17632/2xyyhzts9x.3^(23)^. The statistics were calculated using SPSS version 27.0 statistical software (IBM, Armonk, NY, USA).

## Results

The main finding between the pre- and post-training samples is the increase in the number of Class A CT examinations, which rose from 1.8 to 11.8% resulting in an increased proportion of lens favourable CT examinations.

**Figure 3:**
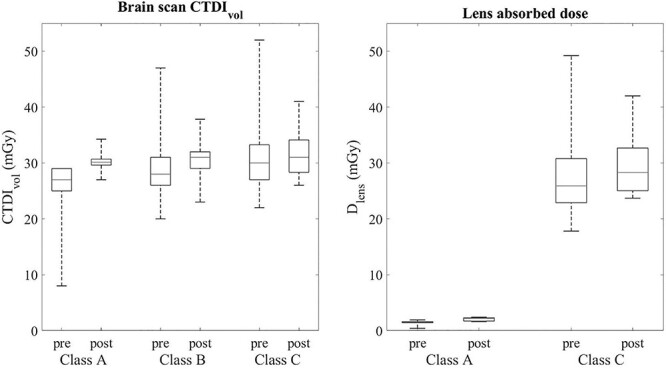
The comparison of CTDIvol and lens absorbed dose pre- and post-training.

**Figure 4:**
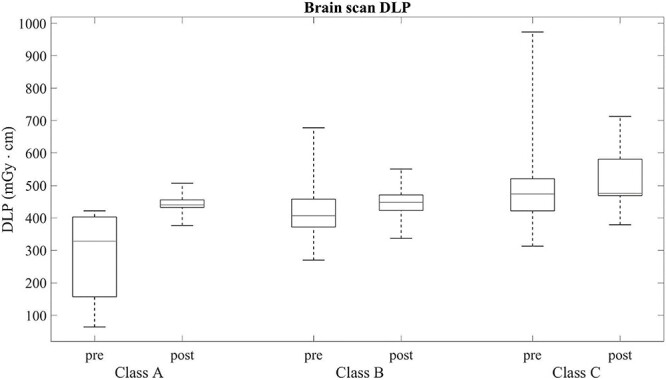
DLP pre- and post-training. The variation of the examination lengths has decreased showing the positive effect of training in examination quality. In Class A, the post-DLP has increased as the few patients in it have been scanned consistently from the top of the brain to the SOML.

The ratios of CTDI_vol_ and the lens doses are detailed in Table 1. The CTDI_vol_ tended to be higher in CT scans where lens is in the imaging area. The absorbed dose of the lens (D_lens_) was 25.9 mGy in Class C and 1.5 mGy in Class A in the pre-training setting and, respectively, 28.3 mGy in Class C and 2.2 mGy in Class A in the post-training setting (Figure 3). The DLP values also tended to be higher in the Class B and C examinations compared with the Class A classified examinations (Table 1 and Figure 4).

**Table 1:** Medians and ranges for the CTDI_vol_, DLP and lens doses. *P*-values represent the statistical difference between the Class A–C groups.

**Pre-training**				
	Class A	Class B	Class C	*P*-value
*n* (%)	6 (1.8)	226 (68.7)	97 (29.5)	—
CTDI_vol_ mGy (median and range)	27 (8–29)	28 (20–47)	30 (22–52)	<0.001^a^^)^
DLP (median and range)	329 (64–422)	407 (270–678)	474 (313–973)	<0.001^a^^)^
D_lens_ mGy (median and range)	1.5 (0.4–1.9)	—	25.9 (17.8–49.2)	<0.001^b^^)^
D_lens_/CTDI_vol_ (median and %)	5.6 (5.0–6.8)	—	87.3 (65.9–129.4)	—
**Post-training**				
*n* (%)	6 (11.8)	30 (58.8)	15 (29.4)	—
CTDI_vol_ mGy (median and range)	30 (27–34)	31 (23–38)	31 (26–41)	0.787^a^^)^
DLP (median and range)	441 (377–507)	448 (337–551)	476 (379–713)	0.003^a^^)^
D_lens_ mGy (median and range)	2.2 (1.6–2.4)	—	28.3 (23.7–42.0)	<0.001^b^^)^
D_lens_/CTDI_vol_ (median and %)	6.7 (5.3–7.7)	—	95 (69.7–123.5)	—

^a^Kruskal–Wallis test.

^b^Mann–Whitney *U* test.

In our study, the absorbed dose to the lens was 66–129% of CTDI_vol_ in cases where the lens is fully exposed to the primary radiation. The lens dose decreased considerably when the lens were cropped from the scanning area as the absorbed dose to the lens was 5–8% of CTDI_vol_.

**Figure 5:**
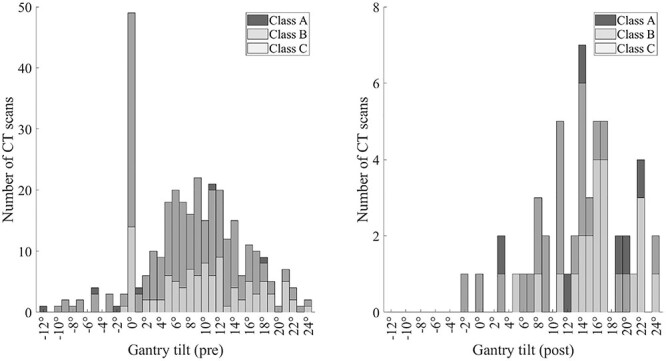
Histogram of the used gantry tilt angles before (left) and after (right) training. The distributions are similar but the extensive use of 0-degree tilt angle is not present after training.

According to the guidelines the head should be tucked (flex the neck to bring the chin towards the sternum) and/or the gantry tilted to fit the angle to the SOML^(24)^, which is verified from the scout image. The most frequent gantry angle found in the DICOM headers was 0° (Figure 5) in the pre-training setting, as the radiographers are instructed to use zero angle when the patient’s head is tucked and the SOML is vertical. The rest of the gantry angles were approximating normal distribution with average value of 9.5°, indicating that some tilt is usually required since the patient’s head is not perfectly tucked. In the post-training setting, the most frequent tilt angle was 14°. The proportion of examinations with gantry tilt used did differ in pre- and post-training setting. Gantry tilt was used in 85% of examinations in pre-training setting and 98% of examinations in post-training setting (Figure 5).

## Discussion

CT has become a crucial tool in medical diagnostics, which explains the steep increase in its use during the past two decades. The radiation exposure can be justified by the benefits of the exam to an individual patient, but in the population aspect there is a concern about rising radiation doses. Over past years, the reduction of the radiation doses has been an important technology driver and lots of improvement have already been achieved. Important dose reduction has been obtained by more efficient X-ray beam collimation and advanced reconstruction algorithms. Although having significantly decreased during the last decade, X-ray dose is still a major concern of patients receiving a CT scan. The amount of exposure still depends on the daily imaging practices and optimisation. While the number of CT examinations is growing, the optimisation efforts also need to increase. In the daily hospital practice, the rising trend in CT imaging is continuing especially in the emergency setting^(25)^, which makes the practice performance even more challenging. It is typical that there are CT equipment of different manufacturers and models in the same hospital, all of which do not enable gantry tilt. Radiographers often use several different CT scanners in their busy daily routine. So, radiographers should have strong expertise and knowledge with each device in order to use the features of each device as efficiently as possible and the optimisation is high quality.

In this two-part study, we found that the training of radiographers increases the quality of the CT examinations. However, even after the training, only 11.8% of the examinations were optimal. When we further investigated individual CT examinations, especially in the post-training sample, it was clear that it may not be possible to include the base of the skull and exclude the lens at the same time even if gantry tilt is used. This is because the neck mobility of some patients was so limited that the gantry could not be tilted enough. In addition, as many patients are brought on CT scans as trauma patients, some may have spinal immobilisation devices that exclude the possibility to tilt the neck. Because the lens cannot always be completely excluded, perhaps in some cases it is better to include a few extra slices, even if the vitreous is partially in the imaging area, to ensure that the posterior parts of the brain are included in the imaging area. If the posterior parts of the brain were left out of the imaging area, it would lead to additional CT scans and to a much higher radiation exposure.

Previously bismuth shields have been used to protect the lenses. Perisinakis *et al*.^(26)^ studied the effect of using bismuth shielding in reducing the lens dose. They stated that bismuth shielding can reduce the lens dose by 38% when the whole orbital area is scanned. Several studies have shown that gantry tilt is more effective in reducing the eye lens dose compared with bismuth shielding^(19, 27)^. In addition, shield use is counterproductive as the increased attenuation in the orbital area causes the device to increase tube current in that area leading to increased lens dose. The recent European consensus of patient contact shielding^(28)^ and AAPM Position Statement do not recommend to use bismuth shielding for eye lens dose reduction^(29)^. Some studies have investigated the organ-based tube current modulation techniques to reduce the lens dose. The reported lens dose reduction varies from 9 to 30%^(30–32)^. Based on the literature and our findings, it seems that the tilting of gantry remains the most effective way to reduce the eye lens dose in CT examinations^(33)^.

We chose to study the lens dose optimisation at our Revolution GSI scanner because it features tilting gantry and because it is used to perform most of the elective CT examinations of the brain at our hospital. We wanted to focus on elective studies to minimise distractions and rush often related to emergency examinations. There are a total of six CT scanners in the Tampere University Hospital and its satellites, and a total of 15 000 brain CT scans are performed yearly. The patient volume is distributed between the scanners based on availability, scanning features, dose and image quality. We chose to include only patients of age 16–40 in the retrospective part of this study because they may receive high cumulative dose from X-ray examination during their remaining lifetime. This limits the number of included examinations because a vast majority of CT scans of the brain are conducted on patients older than 40. We did not include children under the age of 16 because different imaging protocol is used for them.

In the pre-training setting, the most used gantry tilt was 0-degrees. After the training, the usage of 0-degree gantry tilt dropped to almost zero. This may indicate that more attention was paid to optimise the studies after the training.

A study carried out in Iran in 2020 by Ebrahiminia *et al.*^(16)^ found that the training of the radiological technologists resulted in the reduction of radiation dose to the lens by 83%. In our study, we could not calculate a similar percentage, since the dose estimations for the Class B studies could not be calculated. However, our CT-Expo dose estimations suggest that if the gantry tilt is adequate, a similar dose reduction can be achieved. Suzuki *et al.* used radio-photoluminescence glass dosemeters to measure the lens dose and found that the mean ratios of lens dose to CTDI_vol_ varied between 81 and 103% at different scanners, when the lens was completely in the scan area. In our study, the ratio varied between 66 and 129%. Suzuki *et al*.^(34)^ also found that when the SOML is used for imaging, the actual radiation dose to the lens is about 10– 18% of the CTDI_vol_ value given by the CT device. In our study, the ratio of lens dose to CTDI_vol_ was 7–17% when the SOML was used for imaging. Our results are in good agreement with Suzuki’s, even though GE Revolution GSI scanners were not included in their study.

There were a few limitations in the dose modelling in our study. First, gantry tilt could not be modelled in CT-Expo. However, this limitation does not markedly change the results as in the modelled cases the lenses were either fully inside the primary radiation field or completely outside it. Second, instead of modelling *Z*-direction tube current modulation, the tube current selected from the slice at the middle of the lens was used in the calculations. CT-Expo supports current modulation; however, the control over how the current is precisely modulated in the lens area was lacking. It was important to select the used tube current in the lens area to produce realistic results of the dose. The tube current modulation outside the lens area may cause negligible changes in the dose because of scattered radiation. The current modulation was tested in CT-Expo and it did not significantly change the overall results. Because of that and the lack of control, static current was chosen. Third, lens doses in Class B were not modelled because of the resolution of the phantom in CT-Expo. The doses in Class B may be lower than the doses in Class C, but not as low as in Class A as the lens was partially exposed. Fourth, standard adult female and male phantoms were used in modelling the lens doses. Because only the eye-lens doses were of interest the calculations were restricted to the head. The natural variation in the body composition of the sample has a minor effect in the head area. Therefore, the use of the standard phantoms does not compromise the conclusions. Fifth, the number of patients in Class A is much lower than in Classes B and C. This was the distribution found in the study which reflects the level of optimisation. The modelled doses were a decade lower in Class A than in Class C, and thus, it would be unlikely that a higher *n* in Class A would change the magnitude of the results.

## Conclusions

In conclusion, eye lenses are irradiated in brain CT examinations and as they are not of primary interest in the scan, the dose should be reduced according to ALARA. Optimal use of gantry tilt and scan length reduces the lens doses significantly. Radiographer training can save lens doses in the population level. Radiation safety in imaging requires continuing effort where the imaging protocols are optimised, and personnel trained to keep the current level and incrementally improve through development projects and new technology.

## Funding

This work has not been supported by funding agencies. All the authors have worked in the Tampere University Hospital when the study has been conducted.
